# The body mass index is associated with increased temporal variability of functional connectivity in brain reward system

**DOI:** 10.3389/fnut.2023.1210726

**Published:** 2023-06-14

**Authors:** Yiqun Guo, Yuxiao Xia, Ke Chen

**Affiliations:** ^1^School of Innovation and Entrepreneurship Education, Chongqing University of Posts and Telecommunications, Chongqing, China; ^2^Research Center of Biomedical Engineering, Chongqing University of Posts and Telecommunications, Chongqing, China; ^3^The Second Affiliated Hospital of Guangzhou Medical University, Guangzhou, China

**Keywords:** obesity, body mass index, resting-state connectivity, temporal variability, reword network

## Abstract

The reward system has been proven to be contributed to the vulnerability of obesity. Previous fMRI studies have shown abnormal functional connectivity of the reward system in obesity. However, most studies were based on static index such as resting-state functional connectivity (FC), ignoring the dynamic changes over time. To investigate the dynamic neural correlates of obesity susceptibility, we used a large, demographically well-characterized sample from the Human Connectome Project (HCP) to determine the relationship of body mass index (BMI) with the temporal variability of FC from integrated multilevel perspectives, i.e., regional and within- and between-network levels. Linear regression analysis was used to investigate the association between BMI and temporal variability of FC, adjusting for covariates of no interest. We found that BMI was positively associated with regional FC variability in reward regions, such as the ventral orbitofrontal cortex and visual regions. At the intra-network level, BMI was positively related to the variability of FC within the limbic network (LN) and default mode network (DMN). At the inter-network level, variability of connectivity of LN with DMN, frontoparietal, sensorimotor, and ventral attention networks showed positive correlations with BMI. These findings provided novel evidence for abnormal dynamic functional interaction between the reward network and the rest of the brain in obesity, suggesting a more unstable state and over-frequent interaction of the reward network and other attention and cognitive networks. These findings, thus, provide novel insight into obesity interventions that need to decrease the dynamic interaction between reward networks and other brain networks through behavioral treatment and neural modulation.

## 1. Introduction

Long-term sedentary office and high-calorie food consumption lead to excessive fat accumulation, which has become a primary contributing reason for weight gain in modern society (1). WHO criteria define overweight in adults as a body mass index (BMI) of 25–29.9 kg/m^2^ and obesity as a BMI of 30 kg/m^2^ or higher (2). Obesity is the biggest worldwide health problem and is associated with chronic diseases such as type 2 diabetes, stroke, cancer, depression, and anxiety (3). More than 1.9 billion adults are overweight, among whom 650 million are obese. If these trends continue, global obesity prevalence will surpass 18% in adults by 2025 (4). However, treatments for obesity have been sub-optimal, which is partly because understanding of its neurobiological correlates remains to be limited.

Obesity and related overeating are associated with hyperactivity in the limbic network (LN) such as the orbitofrontal cortex [OFC (5)], which is hedonically driven and involved in the control of food intake, even in the presence of satiety (6). Relative to those with normal weight, obese people had higher responses to the limbic reward system and its connections to visual food cues (7, 8). Resting-state functional magnetic resonance imaging (rs-fMRI) can measure the intrinsic functional organization of the brain, of which functional connectivity (FC) is widely used as an indicator of synchronization between regions (9). A number of rs-fMRI studies showed that BMI was associated with abnormal FC across networks, such as LN, attention network, sensory motor network (SMN), default mode network (DMN), and frontoparietal network (FPN) (10–12). Nevertheless, some studies did not find these correlations (13, 14). One possible reason for these inconsistent results is that the majority of earlier studies have applied a “static” FC, which ignored the variability of FC between regions over time (15).

Using dynamic, rather than static, connectivity analysis could best explain the variability of neurobiological correlates in obese individuals (16). It sheds new insights on the dynamic spatiotemporal organization of resting brain activity and captures FC related to obesity. Recently, some studies adopt this method to capture FC abnormality related to obesity. For instance, Tan et al. (17) found that obese individuals showed disrupted dynamic FC between basal ganglia and salience network involving visceral sensory and autonomic information. In addition, Park et al. (16) found that abnormal obesity showed aberrant dynamic FC across different networks including FPN, SMN, DMN, basal ganglia, and visual network (VN). However, these studies used the k-means clustering method to investigate the connectivity state changes of the whole brain, ignoring the dynamic connectivity profile of particular brain regions and networks (18).

A recent approach allows to measure the temporal variability of FC of a specific brain region and network over time and reflects the flexibility and adaptability of brain function, which have been applied in many diseases by showing significant variability changes between groups and regions, showing significant variability correlated with behavior (18–20). This method provides a dynamic insight into the understanding of the underlying neuroimaging basis of obesity. To date, evidence on the temporal variability of FC of individual differences in BMI remains limited. This approach can fill this knowledge gap to reveal the abnormality of regional and network-level dynamics of functional connectivity related to BMI. In this study, we aimed to investigate the relationship of BMI with the temporal variability of FC at integrated multilevel perspectives (regional, intra-network, and inter-network), in adults from the Human Connectome Project (HCP) dataset. First, we constructed the temporal variability of regional FC architecture (20). Similarly, the within- and between-network temporal variabilities of FC architecture were constructed using the method introduced by Sun et al. (19). Linear regression analysis was used to investigate the association between BMI and temporal variability of FC, adjusting for covariates of no interest. Given that LN involved in reward processing contributing to the vulnerability of obesity and that obese individuals showed higher FC in reward-related regions (21), we hypothesized that BMI was positively associated with the regional and network-level variability of FC in LN.

## 2. Materials and methods

### 2.1. Participants

Participants were selected from the 1,200 Subjects Release of the Human Connectome Project (HCP) from the Washington University–University of Minnesota (WU–Minn HCP) Consortium (22). Detailed information about the HCP database is provided in the 1,200 Subjects Data Release Reference Manual (https://www.humanconnectome.org/, accessed on 10 March 2021). In this study, the exclusion criteria of participants indicated as follows: (1) participants with missing demographic variables such as age, sex, education, and race or family information; (2) participants with a history of hyper/hypothyroidism or other endocrine problems; (3) women who had recently given birth; and (4) participants with mean frame-wise displacement [FD (23)] >0.25. To this end, we obtained a total of 954 participants from 428 families. Considering the BMI is highly heritable [ranging from 0.47 to 0.90 (24)], we randomly selected one participant from one family to eliminate the heritability influence, as conducted by other studies, employing the HCP dataset (25). Finally, 428 participants for which fMRI images and BMI were used for the current analyses were included (see Table 1 for details). Full informed consent from each participant was obtained by WU–Minn HCP Consortium, and research procedures and ethical guidelines were followed in compliance with WU institutional review board approval.

**Table 1:** Demographic characteristics of participants^a^.

**Variable**	
Body mass index (BMI), mean (SD), kg/m^2^	25.86 (4.41)
Age, mean (SD), years	28.61 (3.75)
Female (Sex), *N* (%)	223 (52.10)
Education, mean (SD), years	14.86 (1.80)
Handedness, mean (SD)^b^	64.65 (45.24)
**Race** ***N*** **(%)**	
White	310 (72.43)
Other	118 (27.57)

^a^Further definitions are available at the Human Connectome Project Data Dictionary.

^b^Handedness of participants from −100 to 100 is assessed using the Edinburgh Handedness questionnaire.

### 2.2. MRI scanning protocols

Rs-fMRI data were collected in four runs of ~15 min, each on a Siemens 3T Tim Trios MRI scanner using the multi-band EPI pulse sequence. For the maximum number of available data, only the left-to-right phase encoding direction was utilized (26, 27). During scanning, participants were required to keep their eyes open with relaxed fixation, think of nothing, and not fall asleep. The rs-fMRI scanning parameters were as follows: a resolution of 2 mm^2^ isotropic; TR = 720 ms; TE = 33 ms; flip angle = 52°; FOV = 208 × 180; 72 slices. T1-weighted images were collected by using the MPRAGE sequence with the following scanning parameters: TR = 2,400 ms; TE = 2.14 ms; flip angle = 8°; FOV = 224 × 224; voxel size = 0.7 × 0.7 × 0.7 mm^3^; 256 slices.

### 2.3. MRI preprocessing

Rs-fMRI data were preprocessed by the minimal preprocessing pipeline, including *fMRIVolume* and *fMRISurface pipelines*. The first pipeline removed spatial distortions, realigns volumes to compensate for subject motion, registers the fMRI data to the structural, reduces the bias field, normalizes the 4D image to a global mean, and masks the data with the final brain mask. The second pipeline aimed to take a volume timeseries and map it to the standard CIFTI grayordinate space used for subsequent resting-state analyses [see Glasser et al. (28) for more details]. To reduce the biophysical noise, we regressed our linear trend and further used CompCor to regress out nuisance covariates including five principal components of white matter and cerebrospinal fluid signals, and Friston 24 head motion parameters. Volumes that FD exceeded 0.5 mm were scrubbed. All images were filtered using a band-pass filter [1/*w*−0.1 Hz, high pass filtering using 1/*w* is suggested to remove spurious fluctuations in dynamic FC, when a certain window size *w* is given (29)].

### 2.4. Temporal variability of FC

#### 2.4.1. Regional-level variability

We used the 300 ROI set from Greene lab as nodes of the whole brain functional network (30) because it further added additional nodes from subcortical and cerebellar structures based on Power 264 atlas (31). To characterize the temporal variability of a specific brain region, all BOLD time series were first segmented into *n* overlapping windows with length *l* (19, 20, 32). Within the *i*th window, a *q* × *q* Pearson correlation matrix (*q* = the number of nodes) describes the FC architecture of the whole brain (*Fi*). The FC architecture of ROI *k* at time window *i* is denoted by *F*(*i,k*), which represents the whole-brain functional architecture of region *k*. Then, the variability of FC architecture for a brain region *k* is defined as follows:


$$\begin{array}{l} V_{k}=1-\bar{corrcoef\left(F\left(i,\;k\right),\;F\left(j,\;k\right)\right)},\;i,\;j\;=1,\;2,\;3,\;\cdots \;,\;n;\;i\neq j \end{array}$$

Similarly, we computed *V*_*k*_ at different window lengths [*l* = 20, 22, 24, … 40 s, (19, 20, 32)] and then took the arithmetic average value as the final variability to avoid the arbitrary choice of time window length. Notable, higher *V*_*k*_ of a region indicates that more functional communities of this region will be involved across time (20).

#### 2.4.2. Within- or between-networks variability

In order to assess the dynamic interactions within- and between-networks, we divided the 300 ROIs into nine prior brain networks, which are consisted of seven networks defined by Yeo et al. (33), including VN, dorsal attention network (DAN), ventral attention network (VAN), SMN, FPN, DMN, and LN. The basal ganglia and cerebellum were treated as a single network, given it was poorly defined into different resting-state FC networks as well as its special role in understanding pathophysiologic mechanisms in obesity (34, 35). Then, we defined variability of functional architecture within- or between-networks in a similar method adopted in the regional variability above (19). For a given brain network *m*, all FCs within this network in window *i* were reshaped as 1D vector, *F*_*mi*_; similarly, for all FCs' between-network, *I* and *p* in window *i* were denoted as 1D vector, *F*_*mi, lmi, p*_. Then, the variability of FC architecture within-network *m* across *n* windows (which is shortened as within-network variability in the follow-up sections) is defined as follows:


$$\begin{array}{l} V_{w_{m}}=1-\bar{corrcoef\left(F_{mi},\;F_{mj}\right)},\;i,\;j\;=1,\;2,\;3,\;\cdots \;,\;n;\;i\neq j \end{array}$$

The variability of FC architecture between-network *l* and *p* is defined as follows:


$$\begin{array}{l} V_{bl_{p}}=1-\bar{corrcoef\left(F\left(mi,\;lmi,\;p\right),\;F\left(mj,\;lmj,\;p\right)\right)},\\ i,\;j\;=1,\;2,\;3,\;\cdots \;,\;n;\;i\neq j \end{array}$$

A high value of within- or between-network variability means the FC architecture within the network, or the interaction between networks, has frequent information communication across different time windows but does not maintain a stable pattern (19).

### 2.5. Statistical analysis

For each regional node or network, the association between the variability of FC and BMI was investigated using linear regression analyses. We first examined the relationships between BMI and basic demographic variables. We found that BMI did not show a significant correlation with age (*r* = 0.0009, *p* = 0.85), sex (*t* = 1.86, *p* = 0.063), handedness (*r* = −0.077, *p* = 0.113), and race (*t* = 0.261, *p* = 0.795). However, BMI was significantly correlated with years of education (*r* = −0.165, *p* = 0.001). To rule out the potential effect of these basic demographic variables on the relationship between BMI and dynamic FC, age, sex, years of education, handedness, race (categorized as white or other), and mean FD were considered as covariates of no interest in the regression model. Considering the distribution of BMI did not follow the normal distribution (Kolmogorov–Smirnov test, *p* < 0.05), we used a permutation analysis of linear models (36), to determine the significance for all association analyses. The fundamental advantage of permutation inference is its reliance on weak assumptions regarding the data. By simply rearranging the observations, a null hypothesis can be tested in a straightforward manner. Despite the existence of nuisance effects or apparent outliers in the data, permutation inference retains its potency and effectively maintains type I error rate control (36). False discovery rate (FDR) correction was used to correct for multiple comparisons (37). We considered an FDR-corrected value of *p* < 0.05 to be significant.

## 3. Results

### 3.1. Demographics characteristic

Demographics and other behavioral characteristics of the final sample are shown in Table 1. The sample comprised 205 men and 223 women, had a mean age of 28.61 ± 3.75 years, was predominantly Caucasian (72.43%), and had a mean BMI of 25.86 ± 4.41 kg/m^2^.

### 3.2. Associations between BMI and variability of regional FC

**Figure 1:**
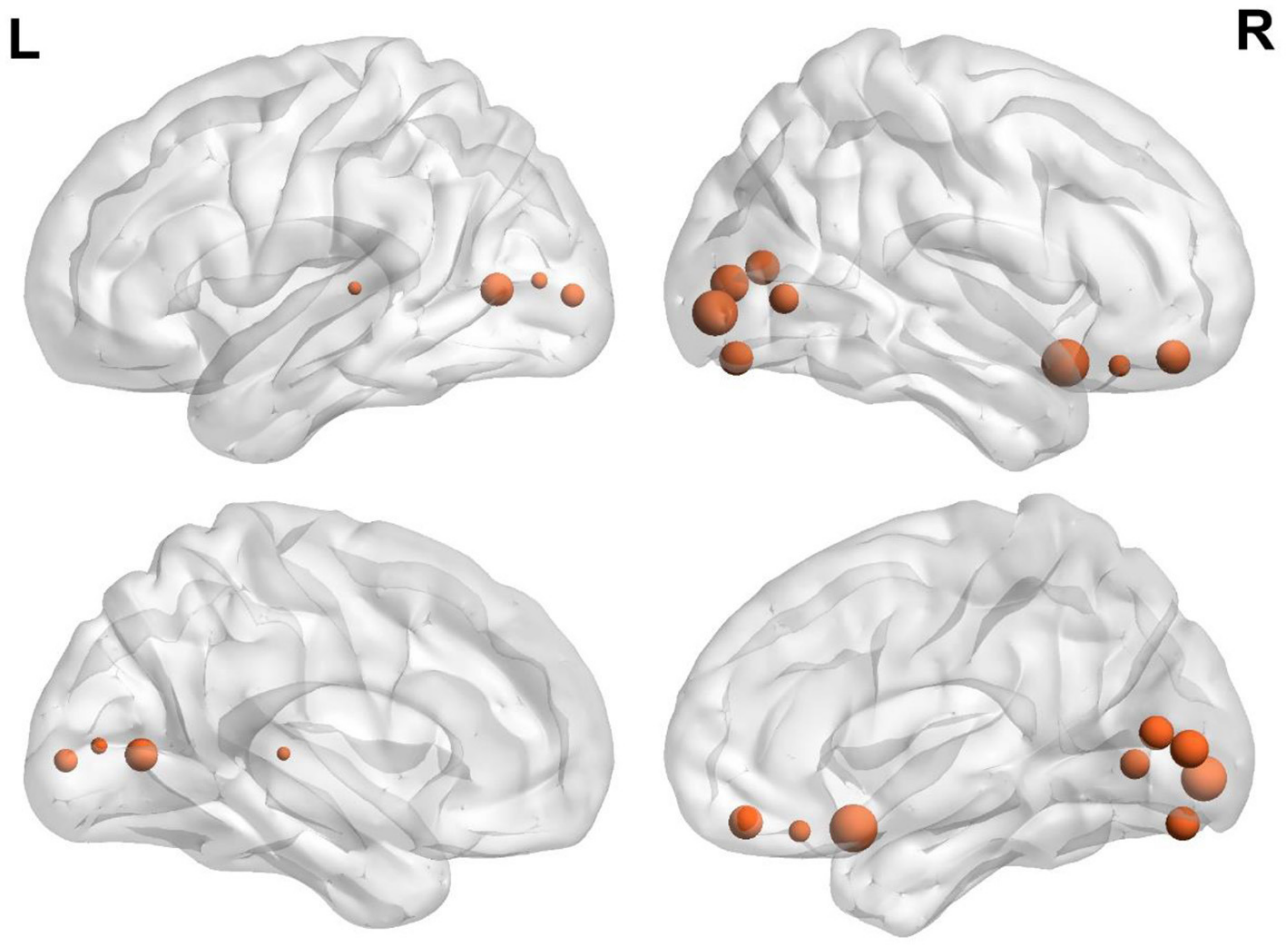
Brain regions demonstrate a significant correlation between BMI and regional temporal variability of FC architecture. The size was weighted by the partial correlation value. All results were shown after FDR corrected (*p* < 0.05).

The variability of FC in ventral OFC and the regions of VN (such as bilateral superior and middle occipital gyrus and lingual gyrus) were found to be positively associated with BMI (Figure 1 and Table 2).

**Table 2:** Significant associations between BMI and variability of regional FC.

**MNI coordinate (** ***X Y Z*** **)**			***r***	***p*-value**	**Network**
23.96	31.94	−17.78	0.157	0.001	Limbic
8.36	47.59	−15.18	0.167	< 0.001	Limbic
27.06	16.22	−16.93	0.178	< 0.001	Limbic
−26.39	−90.23	3.12	0.159	0.001	Visual
−17.87	−68.03	4.81	0.166	< 0.001	Visual
−8.43	−80.5	7.44	0.153	0.002	Visual
6.21	−81.41	6.11	0.170	< 0.001	Visual
8.45	−71.84	10.79	0.167	< 0.001	Visual
19.81	−65.56	1.72	0.163	< 0.001	Visual
19.64	−85.62	−2.39	0.176	< 0.001	Visual
25.66	−79.47	−15.56	0.167	< 0.001	Visual
−49.14	−26.3	5.18	0.151	0.002	Sensorimotor

### 3.3. Associations between BMI and within-network variability of FC

**Figure 2:**
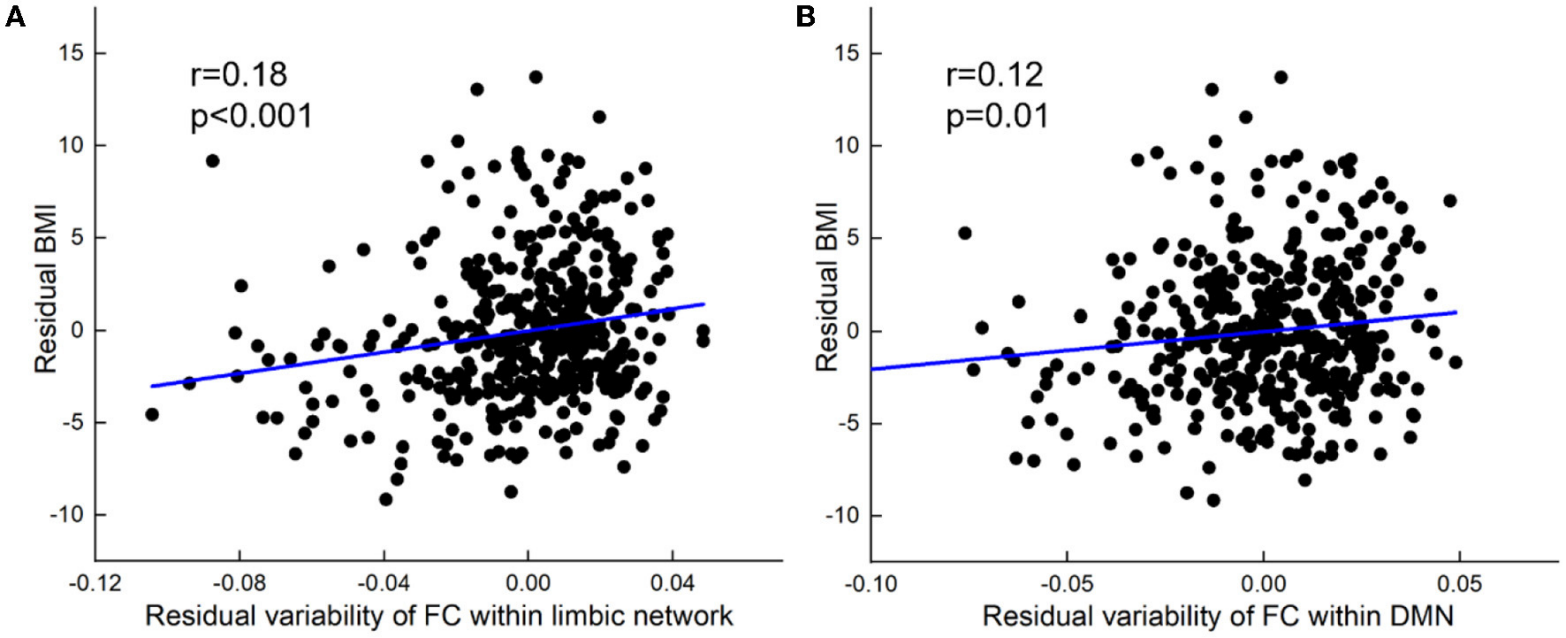
BMI relations to increased variability of FC within network-level. **(A)** Positive correlation between BMI and variability of FC within the limbic network. **(B)** Positive correlation between BMI and variability of FC within DMN. All results were shown after FDR corrected (*p* < 0.05).

At the within-network level, BMI was positively related to within-network variability in LN (partial *r* = 0.18, *p* < 0.001) and DMN (partial *r* = 0.12, *p* = 0.01, Figure 2).

### 3.4. Associations between BMI and between-networks variability of FC

**Figure 3:**
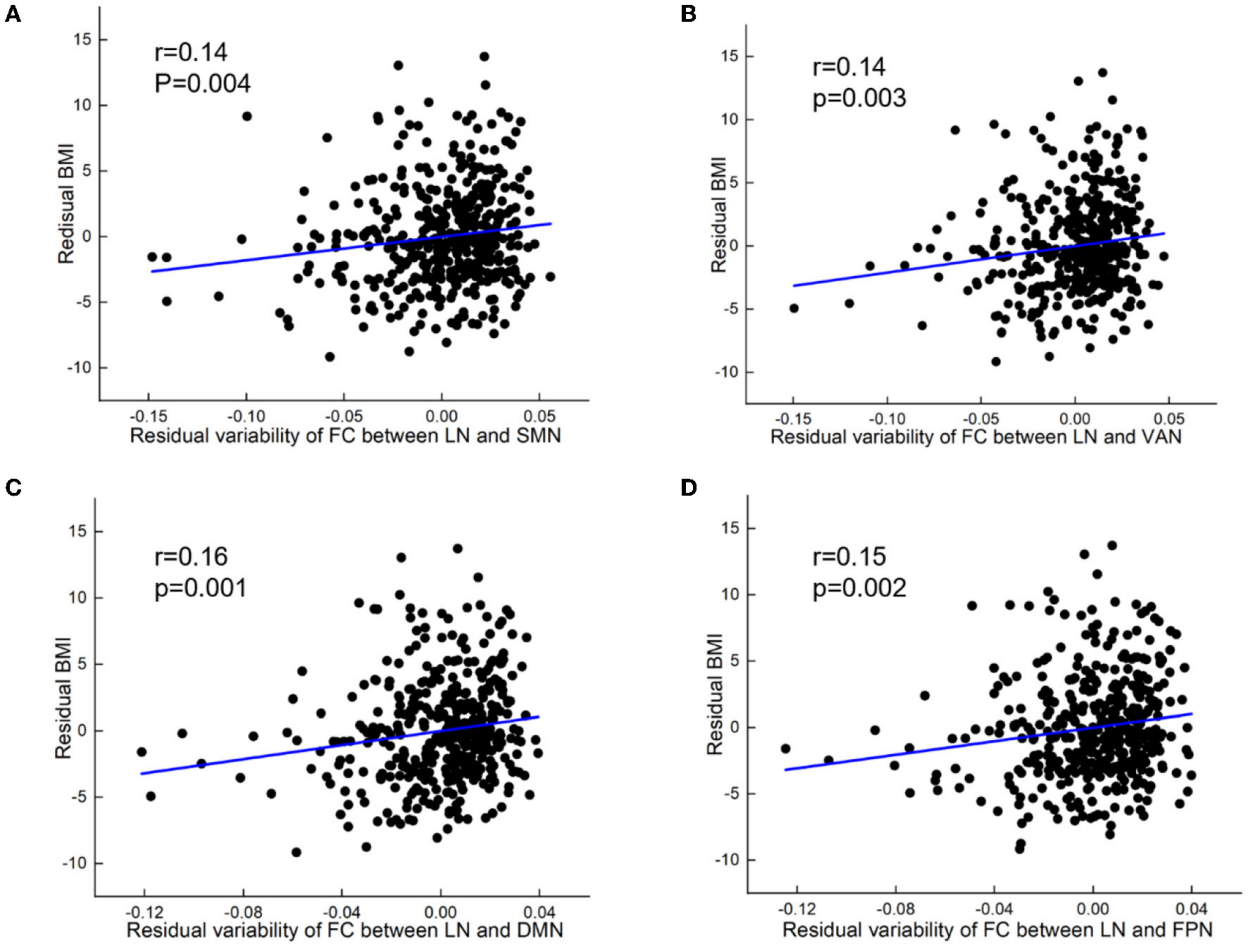
BMI relations to increased variability of FC between networks. **(A)** Positive correlation between BMI and variability of FC between limbic network and sensorimotor network (SMN). **(B)** Positive correlation between BMI and variability of FC between limbic network (LN) and ventral attention network (VAN). **(C)** Positive correlation between BMI and variability of FC between limbic network and default mode network (DMN). **(D)** Positive correlation between BMI and variability of FC between limbic network and frontoparietal network (FPN). All results were shown after FDR corrected (*p* < 0.05).

At the inter-network level, variability of FC between LN and SMN (partial *r* = 0.14, *p* = 0.004), VAN (partial *r* = 0.141, *p* = 0.003), DMN (partial *r* = 0.16, *p* = 0.001), and FPN (partial *r* = 0.15, *p* = 0.002) showed positive correlations with BMI (Figure 3).

## 4. Discussion

In this current study, we investigated the association between BMI and variability of the dynamic functional brain network at regional, within-network, and between-network levels. At the regional level, we found that BMI was correlated with the temporal variability of FC in ventral OFC and visual regions. At the network level, the within-network variability of FC in LN and DMN showed a significantly positive association with BMI. In addition, the between-network variability of FC in LN with FPN, SMN, VAN, and DMN was also positively correlated with BMI. These findings provided novel evidence for abnormal dynamic functional interaction between the reward network (LN) and the rest of the brain in obesity, suggesting a more unstable state and over-frequent interaction of the reward network and other attention and cognitive networks. These findings provided novel evidence for the neurobiology theory of obesity that highlights the critical role of brain regions related to reward in susceptibility to obesity (5). These findings also provide novel insight into obesity interventions that need to decrease the dynamic interaction between reward network and other brain networks through behavioral treatment and neural modulation.

### 4.1. BMI associated with variability of regional FC in ventral OFC and visual regions

The variability of FC architecture in ventral OFC and the regions of VN (such as bilateral superior and middle occipital gyrus and lingual gyrus) was found to be positively associated with BMI. OFC involves in food reward processing and is most important for eating and obesity (12, 38, 39). Occipital and lingual gyrus are important in discriminating high- from low-caloric foods and have greater response to high-caloric foods (40, 41). Neuroimaging studies exploring the relationship between food cue-reactivity and obesity in adults have consistently found reward regions (e.g., OFC) and VN (42–44). Individuals with higher BMI had altered resting-state FC in OFC and visual areas (45), which was correlated with food bias (46). It should be noted that the variability of FC for a given region measures the temporal variability of FC between this given region and the rest of the regions of the brain across time windows. Therefore, higher BMI was linked to greater variability of FC in OFC and visual areas, which possibly indicated that high BMI may be related to more frequent information interaction between regions involving in food reward and attention and the rest of the brain.

### 4.2. BMI associated with within-network variability of FC

We found that BMI was positively related to within-network variability in LN and DMN. This result indicated that FC within LN and DMN network is changing synchronously across different time windows in individuals with higher BMI. Recent studies also found that obese individuals had disrupted dynamic FC in reward and default mode networks (17, 47). It is known that LN is the brain region most associated with food motivation (48, 49). Relative to children with normal weight, obese children had hyper-responsive to food stimuli in LN whether satiety or hunger (50). In addition, a previous resting-state fMRI found that obesity had stronger FC within LN than lean subjects (51). Our result of higher variability of FC in LN revealed that the unstable state of reward function at rest may be the neural correlates of individuals with higher BMI. DMN is a self-reflection and task-negative network that is anticorrelated with areas involved in executive control (52, 53). A previous study found that obese individuals showed altered spontaneous synchronicity within DMN (54). The higher variability of FC within DMN may suggest that abnormal self-integration is associated with higher BMI. Taken together, the higher variability within LN and DMN suggested an unstable pattern within LN and DMN, which provides dynamic neural interaction evidence related to obese vulnerability.

### 4.3. BMI associated with between-network variability of FC

Notably, between-networks variability of LN with FPN, SMN, VAN, and DMN showed a positive correlation with BMI. Similarly, the limbic reward system and its connections showed greater response to visual food cues in obese people, relative to those with normal weight (7, 8). Several neuroimaging studies have been proven that the stable and dynamic connections between these networks represented BMI variability (17, 45, 55–58). Dysfunctional FPN is widely considered the neural basis of obesity and overeating (59), which is indicative of executive control function (16). SMN is considered to govern the translation from goal-directed action to habitual behavior in obese individuals (60, 61). The activation of VAN mainly detects an attention bias to energy-dense and palatable food and over-consumption in disinhibited individuals (62). DMN involves self-reflection and integrating internal and external information (63). Evidence from fMRI studies manifested that the interaction between LN and this network was responsible for food reward processing (64, 65). We speculate these findings that individuals with high BMI may have more frequent information changes between LN and these cortical networks (involved in executive control, habitual behaviors, attention bias, and self-reflection), which further demonstrates that the limbic reward network plays a core role in the vulnerability of obesity from the perspective of temporal variability of FC.

### 4.4. Future directions and limitations

First, this was a cross-sectional study and therefore cannot indicate causal directionality, which requires a longitudinal study to further explore whether alterations in dynamic functional connectivity occur before or after weight gain. Second, this study used self-reported BMI. Future studies may consider the use of a medical body composition analyzer to measure BMI, which is more accurate than self-reported. Third, although several key covariates were considered in the analyses, the possible influence of other unmeasured variables such as genetics and personality on associations cannot be ignored. Finally, all participants in the study were selected from a young adult group. Caution is needed when generalizing our findings.

## 5. Conclusion

This current study reported alterations of temporal variability associated with BMI at regional, within-network, and between-network levels. Our results showed that high BMI was associated with greater regional variability in the ventral OFC and visual regions and higher temporal variability within LN as well as between LN and FPN, SMN, VAN, and DMN networks. These findings provided novel dynamic neural interaction evidence for the neurobiology theory of obesity that highlights the critical role of the brain system related to reward in susceptibility to obesity, which highlights that obesity interventions need to decrease the dynamic interaction between reward network and other brain networks through behavioral treatment and neural modulation.

## Data availability statement

The datasets presented in this study can be found in online repositories. The names of the repository/repositories and accession number(s) can be found at: https://db.humanconnectome.org/app/template/Login.vm;jsessionid=7D797B490A8CCC42F73CECCCEC8AABCE.

## Ethics statement

The study was approved by the Ethical Committee of Chongqing University of Posts and Telecommunications (protocol code: No. CQUPT2022057 and data of approval: 21 September 2021). The patients/participants provided their written informed consent to participate in this study. Written informed consent was obtained from the individual(s) for the publication of any potentially identifiable images or data included in this article.

## Author contributions

YG: project administration, conceptualization, formal analysis, investigation, methodology, validation, visualization, writing—original draft, and writing—reviewing and editing. YX: writing—original draft and writing—reviewing and editing. KC: writing—reviewing and editing.
